# Heme-Derived Metabolic Signals Dictate Immune Responses

**DOI:** 10.3389/fimmu.2020.00066

**Published:** 2020-01-31

**Authors:** Giacomo Canesin, Seyed M. Hejazi, Kenneth D. Swanson, Barbara Wegiel

**Affiliations:** ^1^Department of Surgery, Cancer Research Institute and Transplant Institute, Beth Israel Deaconess Medical Center and Harvard Medical School, Boston, MA, United States; ^2^Brain Tumor Center and Neuro-Oncology Unit, Beth Israel Deaconess Medical Center, Boston, MA, United States

**Keywords:** heme, biliverdin reductases, inflammation, bilirubin, cancer, liver disease

## Abstract

Heme is one of the most abundant molecules in the body acting as the functional core of hemoglobin/myoglobin involved in the O_2_/CO_2_ carrying in the blood and tissues, redox enzymes and cytochromes in mitochondria. However, free heme is toxic and therefore its removal is a significant priority for the host. Heme is a well-established danger-associated molecular pattern (DAMP), which binds to toll-like receptor 4 (TLR4) to induce immune responses. Heme-derived metabolites including the bile pigments, biliverdin (BV) and bilirubin (BR), were first identified as toxic drivers of neonatal jaundice in 1800 but have only recently been appreciated as endogenous drivers of multiple signaling pathways involved in protection from oxidative stress and regulators of immune responses. The tissue concentration of heme, BV and BR is tightly controlled. Heme oxygenase-1 (HO-1, encoded by *HMOX1*) produces BV by heme degradation, while biliverdin reductase-A (BLVR-A) generates BR by the subsequent conversion of BV. BLVR-A is a fascinating protein that possesses a classical protein kinase domain, which is activated in response to BV binding to its enzymatic site and initiates the downstream mitogen-activated protein kinases (MAPK) and phosphatidylinositol 3-kinase (PI3K) pathways. This links BLVR-A activity to cell growth and survival pathways. BLVR-A also contains a bZip DNA binding domain and a nuclear export sequence (NES) and acts as a transcription factor to regulate the expression of immune modulatory genes. Here we will discuss the role of heme-related immune response and the potential for targeting the heme system for therapies directed toward hepatitis and cancer.

## Introduction

**Figure 1:**
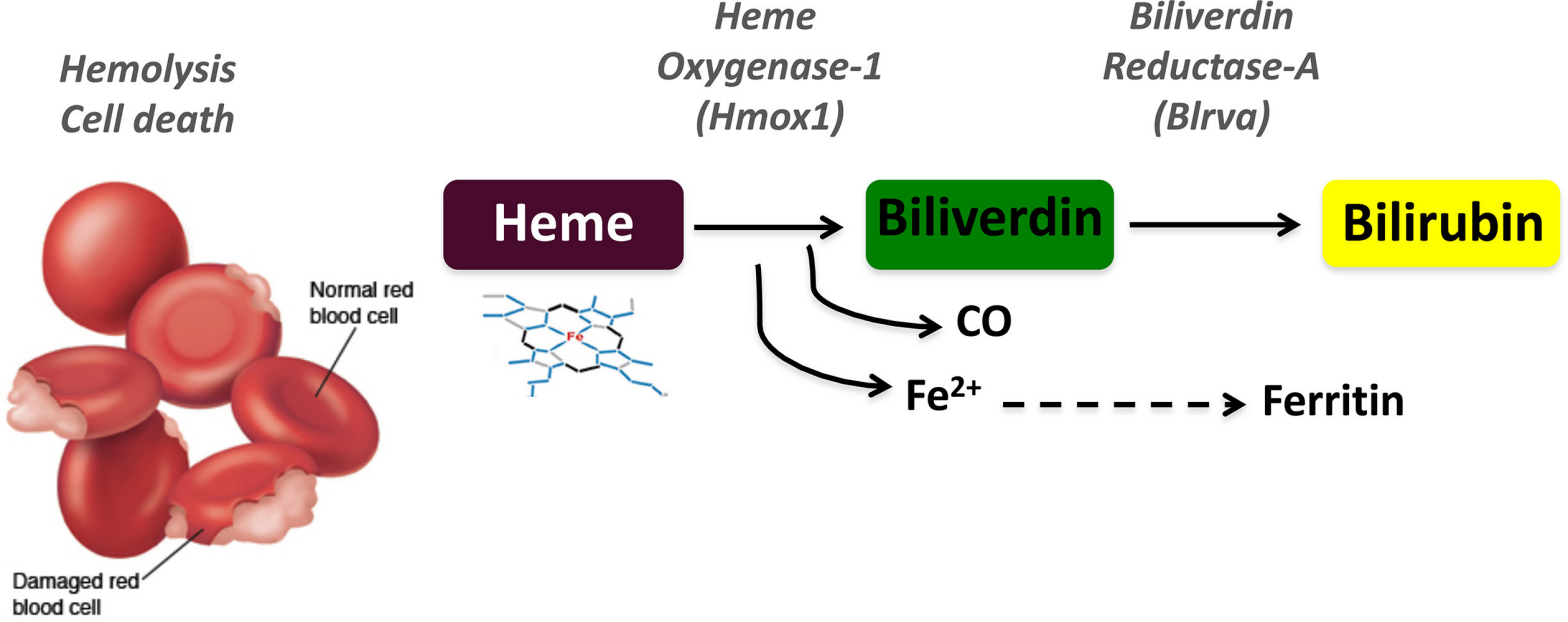
Schematic representation of the heme degradation pathway. Damaged red blood cells release free heme, which is converted to biliverdin by heme oxygenase-1 (HO-1, *HMOX1*) in a reaction that generates carbon monoxide (CO) and iron (Fe^2+^). Biliverdin is in turn converted to bilirubin by biliverdin reductase A (BLVR-A).

Many human diseases are associated with immune dysfunctions affecting the host ability to control inflammation. Under normal physiological conditions, the activation of pro-inflammatory processes is resolved by the controlled response to reinstate tissue homeostasis. For this reason, cells and organisms have evolved to integrate protective molecular mechanisms that specifically counter inflammatory effector functions in order to threshold the response and re-establish homeostasis upon inflammatory resolution (1, 2). The details of heme metabolism have only been appreciated in the last 30 years while bile pigments were known a century earlier. Heme is a tetrapyrrolic porphyrin ring that coordinates an Fe^2+^ atom and is catabolized by heme oxygenases (HO-1, encoded by *HMOX1* or HO-2, encoded by *HMOX2*) into biliverdin (BV), iron, and carbon monoxide (CO) (3–5). The subsequent conversion of BV to bilirubin (BR) is catalyzed by biliverdin reductases (BLVR), for which two isoforms exist in humans, BLVR-A and BLVR-B (Figure 1). These enzymatic reactions can be best visualized by the colorful stages occurring during bruising: (i) the initial dark purple is due to heme release from damaged red blood cells (RBC), (ii) while the green color corresponds to BV, (iii) and finally the yellow color is BR which is also responsible for the yellow pigmentation evident during jaundice.

BV and BR possess strong antioxidant, anti-inflammatory and protective properties (6) and have recently been shown to participate as regulators of inflammatory reactions in sepsis and organ injury. BR exerts general immunosuppressive effects with possibly important clinical consequences, as evidenced by multiple experimental as well as clinical studies (7–11). In humans, BR is the most prominent bile pigment and is very effective at counteracting cellular oxidative stress, protecting cells against lipid oxidation, attenuating oxidative damage to proteins, and acting as a scavenger of nitric oxide (NO) and reactive oxygen species (ROS) (12–14). Consistent with this, mildly elevated plasma BR levels have been negatively correlated with the risk for atherosclerosis, stroke (15, 16), cancer (17, 18), and inflammatory diseases including ulcerative colitis or Crohn's disease (19–23). This strongly suggests an immunomodulatory role for BR. Under physiologic conditions, for disposal from the body, BR is conjugated to glucuronic acid in the liver by bilirubin UDP glucuronosyl transferase (UGT1A1) and then secreted within the bile into the intestinal lumen (24). In the colon, BR is de-conjugated by microbial-produced enzymes and can be partially reabsorbed into the circulation, but it has also been suggested to contribute locally to gut immune homeostasis by modulating T cell activation (25, 26). Furthermore, recent work showed that BR acts as a scavenger of superoxide within neural synapses, protecting against excitotoxicity and neuronal death during neurostimulation with ligands such as with NMDA (27).

The physiological turnover of heme derived from RBC generates both BV and BR. In adults, HO-1-mediated heme cleavage occurs predominantly on the alpha-meso carbon to generate BV. Subsequently, BLVR-A, the predominant BLVR isoform in adults, converts BV to the BR-IXα isoform of BR (28, 29). Conversely, BLVR-B is the dominant isoform found in the fetus and produces the BR-IXβ isoform (30, 31). The highest levels of BR-IXβ are found in fetal bile, indicating that heme catabolism *in utero* differs from that in adults (32). Thus, unconjugated BR-IXβ is the first bilirubin pigment to appear in bile during fetal development, being observed as early as at 14 weeks gestation (33). At 16 weeks gestation, small amounts of unconjugated BR-IXα are also detected in human fetal bile, indicating the maturation of liver-uptake and biliary-secretion mechanisms (33). Furthermore, BR-IXβ accounts for 60–95% of the unconjugated bilirubin in the first sample of excreted meconium, but its amount decreases rapidly during the first 5 days in full-term newborns while declining more slowly in preterm neonates (34, 35). This may be related to the fact that BR-IXβ cannot easily cross the placenta and it needs to be excreted into bile without previous conjugation to glucuronic acid (36).

Heme is the primary inducer of *HMOX1* gene expression. Since the main function of HO-1 is to degrade heme, this results in a negative feedback mechanism for maintaining cellular homeostasis under stress conditions, *HMOX1* expression will be driven in cells and tissues where excess heme is present until the excess heme is cleared (37). HO-1 expression is also induced by other stressors, including UV radiation, hormones, endotoxins, and cytokines. HO-1 exerts anti-inflammatory, anti-apoptotic and anti-proliferative actions in various cell types, including endothelial cells and macrophages (38, 39). This provides a basis for how the heme catabolic pathway may be necessary for preventing tissue injuries in several disease states, from endotoxic shock to ischemia/reperfusion injury, vascular injury, and hepatitis (40–46). Similarly, BLVR are also critical enzymes in the heme catabolic pathway by removing BV. Although BV is a non-toxic molecule, mammalians evolved to remove it within minutes as shown using exogenous administration of BV. Among the reasons why BV is removed is the need for the strong antioxidant BR and/or the necessity to act as a ligand for BLVR-A triggering signaling through PI3K-Akt (47). Functional ligands, as BV, have a short half-life to prevent chronic signaling. Importantly, BLVR-A has been found on the cell surface (BLVR_surf_) where it initiates signaling cascades within the cytoplasm upon extracellular BV-binding (47). BV initiates the activation of tyrosine kinase domain of BLVR-A. Interestingly, BLVR-A possesses dual specificity protein kinase activity (48–50) that plays important roles not only in response to BV (47) but also in the insulin/insulin-like growth factor 1 (IGF1)-signaling pathways, with effects on insulin action, glucose uptake, signal transduction and gene expression (48, 51). Additionally, BLVR-A kinase activity is responsible for the production of IL-10 via PI3K/Akt activation upon binding of BV to BLVR-A in the membrane (29, 47). Through its kinase activity domain, BLVR-A inhibits total glycogen synthase kinase 3β (GSK3β) activity downstream of Akt activation, which supports a role for it in many cellular functions including the modulation of immune response or inflammation regulated by nuclear factor (NF)-κB (NF-κB) (52–54). A recent study by Sharma and colleagues showed that loss of BLVR-A impairs a neuroprotective Akt-mediated inhibition of GSK-3β in response to oxidative stress, thus contributing to early stage Alzheimer's disease (55). However, BLVR kinase activity is dispensable for BLVR-dependent PKC activation. In this settings, BLVR acts as a scaffold to stabilize the active conformation of the PKC (56). This scaffolding role of BLVR may promote the assembly of elaborate signal transduction complexes that facilitate the phosphorylation and subsequent activation of MAPK Erk1/2, either *via* MEK1/2 or *via* PKC (56–58).

**Figure 2:**
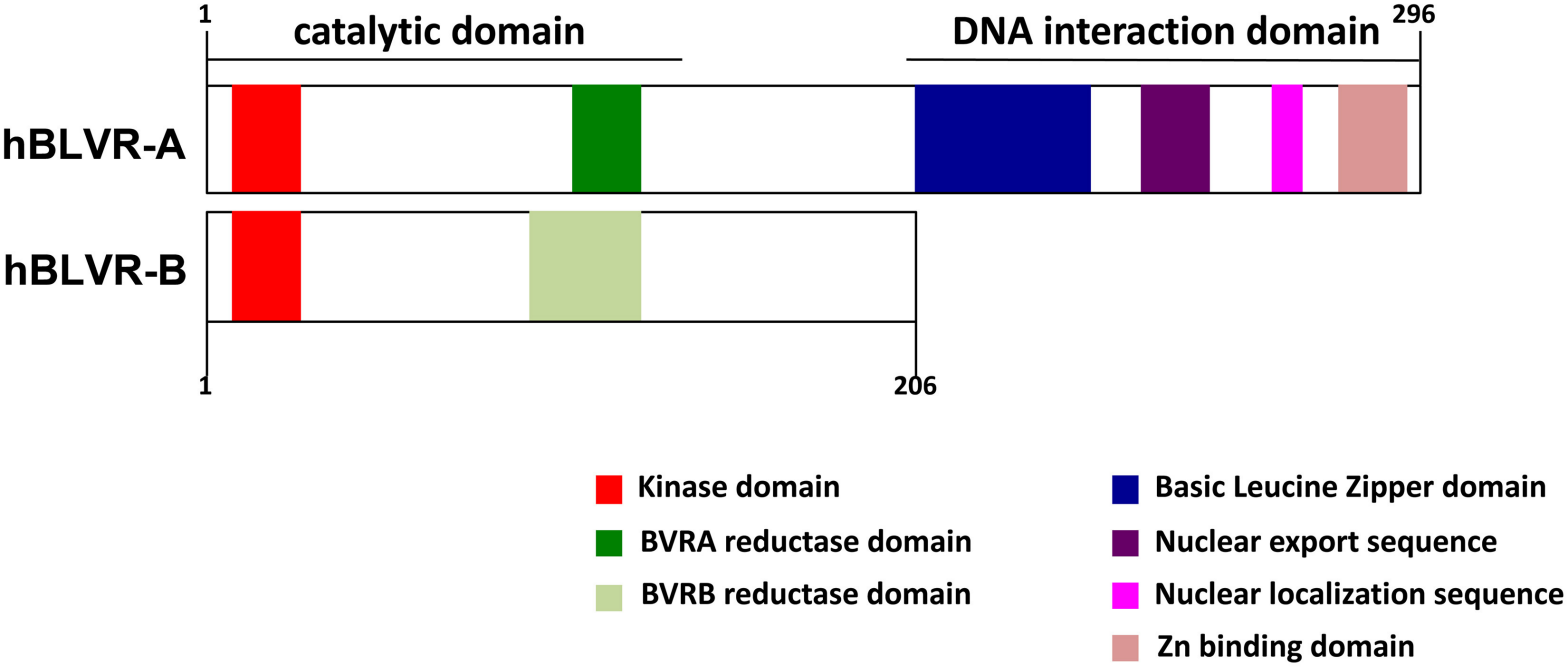
Domain structures of human BLVR-A and BLVR-B. A schematic comparison of the structure of human BLVR-A and BLVR-B shows a similar kinase and catalytic domain for both enzymes. The two isoforms differ in the C-terminus, where BLVR-A contains a bZip DNA binding domain, a nuclear localization sequence and a nuclear export sequence that are not present within BLVR-B.

BLVR-A possesses additional activities and distinct signaling capabilities, which makes it a highly pleiotropic and multifaceted protein (47, 48, 59, 60) (Figure 2). BLVR-A has a direct transcriptional control activity due to a bZip DNA binding domain in its C-terminal domain (61) (Figure 2). Thus, both HO-1 and BLVR act as oxidative stress and inflammatory response enzymes, but also key signaling molecules and are considered to play important roles in response to and protection against cellular stress (62–67). Therefore, understanding the biology of bile pigments and the mechanism of action of BLVR is central to a full comprehension of tissue homeostasis and many immune-associated pathologies.

## HO-1 And Blvr Enzymes: Expression And Modes Of Action

HO-1 is a ubiquitous and evolutionary conserved protein that in mammalian cells localizes mainly to the smooth endoplasmic reticulum (ER) and cytosol, where it primarily degrades heme (68). However, recent evidence suggests an association of HO-1 with other intracellular membranes, including the inner mitochondrial membrane and plasma membrane caveolae (69). HO-1 expression is detected at basal levels in the liver and spleen, which are major sites of iron recycling from hemoglobin (Hb) of senescent erythrocytes phagocyted by macrophages (70). For this reason, liver and spleen tissue macrophages exhibit constitutively high levels of activity and gene expression of *HMOX1* under physiological conditions (71–73). However, *HMOX1* can be induced at the transcriptional level in various other tissues by multiple stimuli, including ROS, heavy metals and its own substrate, heme (74).

**Figure 3:**
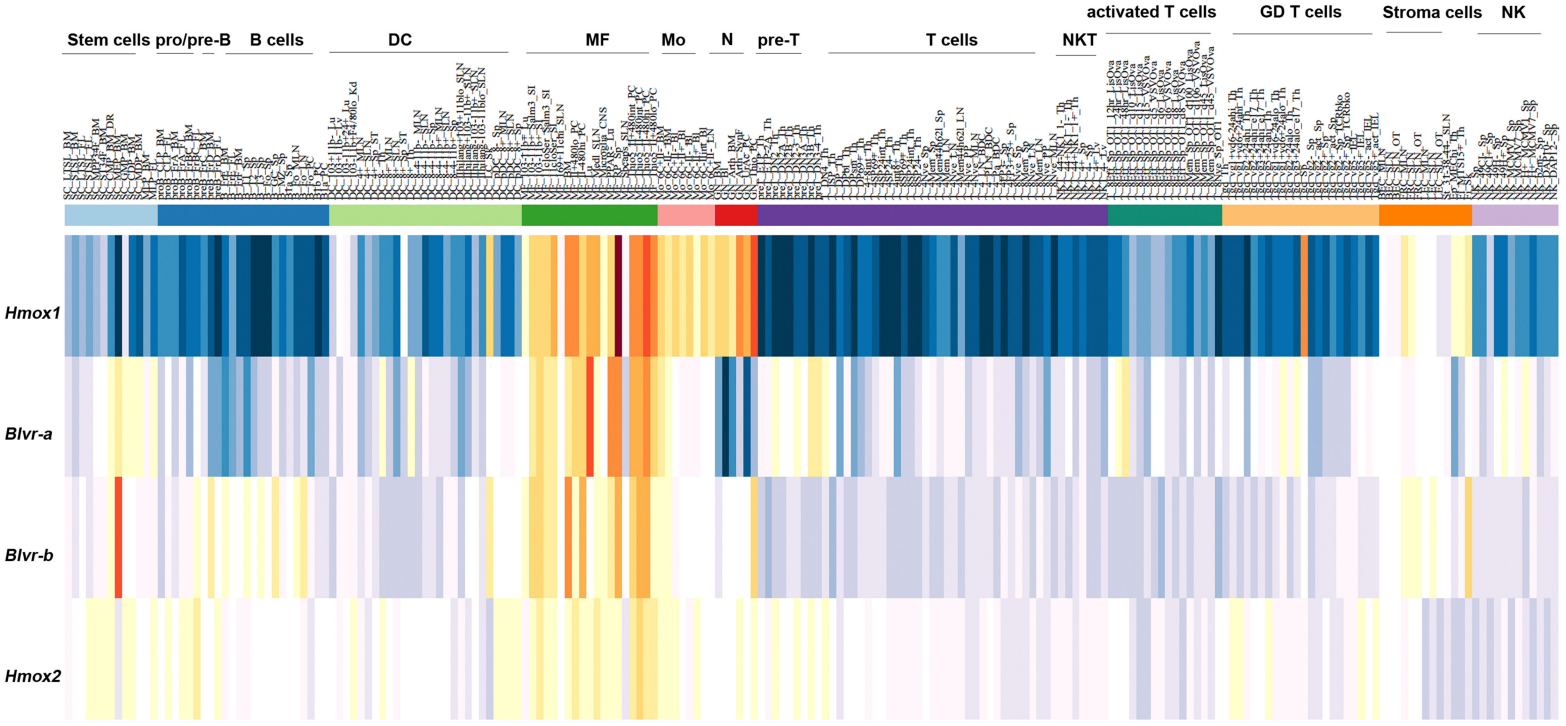
Expression pattern of *HMOX1, HMOX2, BLVR-A*, and *BLVR-B* mRNA in immune cells as reported in the *ImmGen expression data* software (http://rstats.immgen.org/MyGeneSet).

**Figure 4:**
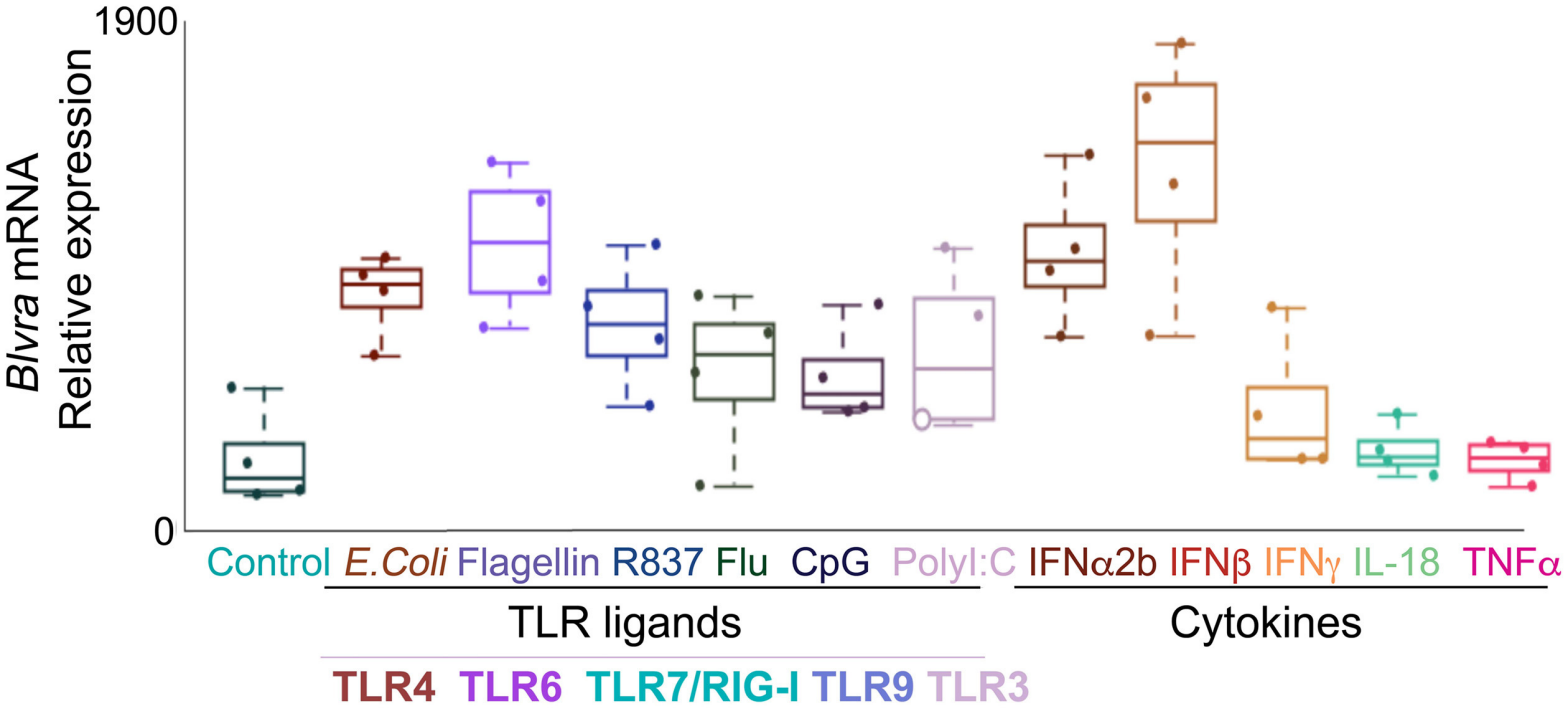
Expression of *BLVR-A* in human leukocytes. qPCR analysis of *BLVR-A* in peripheral blood mononuclear cells after stimulation with toll-like receptor ligands and cytokines. Results represent the mean ± s.d.

*In silico* analysis of the expression of *HMOX1, HMOX2, BLVR-A*, and *BLVR-B* across the *ImmGen expression data* (http://rstats.immgen.org/MyGeneSet) demonstrated that while BLVR-B is generally expressed at highest levels during fetal development, both isoforms of *BLVR* and *HMOX1* are expressed by immune cells, primarily within the myeloid compartment in the adult (Figure 3). *HMOX2* showed less distinct pattern of expression (Figure 3). We and others have reported high levels of *BLVR-A* expression within the reticulo-endothelial system and phagocytic mononuclear populations in the spleen and liver (47, 60, 75). Our previous findings demonstrated that *BLVR-A* expression is also responsive to both LPS and BV treatment in macrophages (29, 76). Additionally, analysis of data obtained from www.immuneprofiling.org in whole blood cells showed that *BLVRA, BLVR-A* is increased in human blood cells following stimulation with various cytokines, DAMPs and pathogen-associated molecular patterns (PAMPs) (Figure 4). The highest induction of *BLVR-A* was observed after treatment with *E. coli*, Flagellin (TLR5 ligand), PolyI:C (TLR7 ligand), R837, influenza virus, IFNα2b or IFNβ. These data suggest a role for BLVR-A in infection with bacteria or viruses. Interestingly, BV seems to interfere with the replication of hepatitis C virus (HCV) by inducing the expression of interferon alpha2 and alpha17, thus triggering an antiviral interferon response (77). A similar antiviral effect has been demonstrated for BR against human herpes simplex virus type 1 (HSV-1) and the enterovirus EV71, indicating that bile pigments and BLVR enzymes can have an important antiviral effect and might improve antiviral therapy (78).

The heme degradation pathway operates in the cytoplasm, where both HO-1 and BLVR are located as either free proteins or associated with endoplasmic reticulum (ER) membranes (79). However, the conversion of BV to BR has been shown to occur in various cellular compartments in response to cellular stress (29, 47, 80). BLVR-A is also found in the nucleus (57, 61), mitochondria (81) and several reports show that its translocation between different cellular compartments is controlled by post-translational modifications such as nitrosylation or phosphorylation (47, 60, 82).

BLVR-A possesses a bZip DNA binding sequence, a nuclear export sequence (NES) and a nuclear localization sequence (NLS) that together enable its bidirectional nuclear transport (Figure 2) (57, 61, 83). BLVR-A is a member of the “leucine zipper” family of transcription factors driving activator protein 1 (AP-1)-regulated genes, such as *HMOX1* (3, 28, 82, 84). BLVR-A can also regulate the expression of genes involved in cell growth, differentiation and survival, which suggests that perturbation of its function may play a role in various disorders and pathologies (48, 84–86).

Since BLVR-B lacks these functions, it is unlikely to possess significant signaling properties or transcription factor activity, representing a major functional difference between the two isoforms (Figure 2) (87, 88). Using a combination of proteomic and transcriptomic approaches, BLVR-B was recently discovered as a novel marker of intraplaque hemorrhage and carotid atherosclerosis, indicating a possible role for its involvement in cardiovascular physiology and disease (89). Interestingly, *BLVR-B* expression was detected in CD163^+^CD68^+^ myeloid cells at both the mRNA and protein levels, but not in lymphocytes, endothelial or smooth muscle cells, which suggests that it somehow controls inflammation in this pathology (89). Thus, both BLVR isoforms play unique roles in the host homeostasis and as such their abnormal expression may lead to pathologies such as autoimmune disease, excessive immune response or cancer.

## HO-1 And Blvr In Inflammatory Responses

HO-1 was initially recognized primarily for its role in heme catabolism and iron recycling, but it has also been described as an enzyme with important anti-inflammatory, anti-oxidant and cytoprotective properties (90). Increased HO-1 expression in tissue is commonly associated with increased inflammation or oxidative stress, as seen in models of acute lung injury or ischemia-reperfusion (91). These anti-inflammatory effects are largely exerted by heme by-products like CO, which exhibits a broad range of immunomodulatory properties in *in vivo* models of endotoxemia, microbial sepsis and organ injury, among others (92, 93). Similarly, HO-1 modulation or application of low concentrations of CO (250 ppm) to cultured macrophages challenged with LPS were shown to reduce the expression of pro-inflammatory cytokines (TNF-α, IL-1β) and simultaneously stimulate the production of the anti-inflammatory cytokine IL-10 through the stimulation of p38 MAPK activity (69, 92, 94). Additionally, previous work demonstrated that macrophage-generated CO can increase ATP release by bacteria, driving the activation of the Nacht, LRR, and PYD domains-containing protein 3 (NALP3) inflammasome in macrophages, thus promoting their bactericidal effector functions (95). Nevertheless, HO-1 up-regulation in macrophages may not always be beneficial: while HO-1 derived CO promotes bacterial clearance by increasing macrophage activity and phagocytosis (96, 97), HO-1 induction has been linked to the intracellular survival of specific pathogens due to reduced inflammatory cytokine production or increased iron availability (98–100). For example, a dual role of heme-derived metabolic signals has been shown in malaria: HO-1 induction amplifies malaria-infection associated liver damage (101), but it also promotes disease tolerance during “the blood stage of the disease” (102). Furthermore, HO-1 induction is known to polarize macrophages into an anti-inflammatory M2 phenotype, which could also be detrimental in infections with pathogens that favor an M2 environment (103). Thus, this duality underlines the complexity of heme catabolism and the role of HO-1 as a critical mediator of innate immune response, indicating that the therapeutic potential of HO-1 may depend on both its expression, its enzymatic activity and the stage of the inflammatory response or disease (104–106).

The role of BLVR-A in inflammation has been primarily described in myeloid cells including macrophages, where this enzyme is expressed as a cell surface protein (47). Both macrophage BLVR-A expression and phosphorylation are increased upon LPS-treatment which leads to anti-inflammatory responses by stimulating PI3K-Akt-driven IL-10 production (47). In the presence of endotoxic stress and induction of NO, macrophage expressed-BLVR-A can also become S-nitrosylated by eNOS (endothelial nitric oxide synthase)-derived NO. This modification leads to its translocation to the nucleus, where it binds directly to the *TLR4* gene promoter and represses its expression (60). These mechanisms have been shown to be important in promoting hepatic diseases such as non-alcoholic fatty liver disease (NAFLD) or non-alcoholic steatohepatitis (NASH), where LPS- and TLR4-associated inflammation plays a significant role (107). NAFLD is characterized by excessive accumulation of fatty acids in the form of microdeposits and its pathophysiology is not fully understood, but insulin resistance and oxidative stress are thought to factor significantly in its progression toward NASH (108). Recent work suggests the regulation of hepatic metabolism by the BLVR-A-GSKβ-PPARα axis where BLVR-A inhibits the phosphorylation of GSK3β resulting in the decreased activation of its substrate, PPARα (109). In this study, mice lacking BLVR-A in hepatocytes showed increased GSK3β activity and PPARα levels, with higher levels of plasma glucose and insulin and reduced glycogen storage (109). Interestingly, BR binds directly to PPARα and elevated total serum BR levels have been reported to negatively correlate with onset of the disease in NAFLD and NASH patients (110–113). These data indicate a major role of BLVR-A in hepatic lipid metabolism and associated inflammation, also supporting an additional role for BR as a protective factor against the progression and development of chronic liver disease. Of note, therapies directed at increasing the activity of BLVR-A or at regulating BR metabolism may prove useful for the treatment of NAFLD (107, 114). A role for BLVR-A in inflammation has also been described in other diseases, such as in germinal matrix hemorrhage (GMH), a neurologic event with high morbidity and mortality in pre-term infants (115). In this model Zhang and colleagues revealed that by suppressing *TLR4* expression, BLVR-A induces the phosphorylation of eNOS in the spleen, modulating the inflammatory response and decreasing neutrophil infiltration into the brain after GMH (115). More recently, a BV/BLVR-A regulatory mechanism that controls TLR4 activation by direct/indirect interaction has been also identified in human leukocytes, suggesting that a fully functional signaling of BLVR could counter undesired TLR4 signaling and related inflammation (116).

BLVR-A has been shown to play a critical role in M2 macrophage polarization both *in vitro* and in response to renal ischemia-reperfusion injury *in vivo* (117). In addition, we have recently shown that BV reduces pro-inflammatory cytokine release and inhibits LPS-mediated C5aR expression in macrophages through the mTOR signaling pathway, further supporting a role for BV as an endogenous anti-inflammatory modulator (75). Moreover, using a mouse mutant for conditional *BLVR-A* deletion in macrophages, we identified a specific set of genes whose expression is altered in the absence of BLVR-A. Among these, the expression of C-X-C motif chemokine 10 (CXCL10 also known as IP-10) and chemokine C-C motif ligand 5 (CCL5, also known as RANTES) was elevated in *BLVR-A* deficient macrophages in response to LPS and this was associated with an increase in C5aR expression and chemotaxis toward C5a (76). Altogether, these studies point to a pivotal role of BLVR-A in controlling the inflammatory response in endotoxemia and beyond, with macrophages and T cells likely participating most in such a mechanism. Interestingly, BR is implicated in the control of T cell function by increasing number of T regulatory cells (26).

## HO-1 And Blvr In Cancer

**Figure 5:**
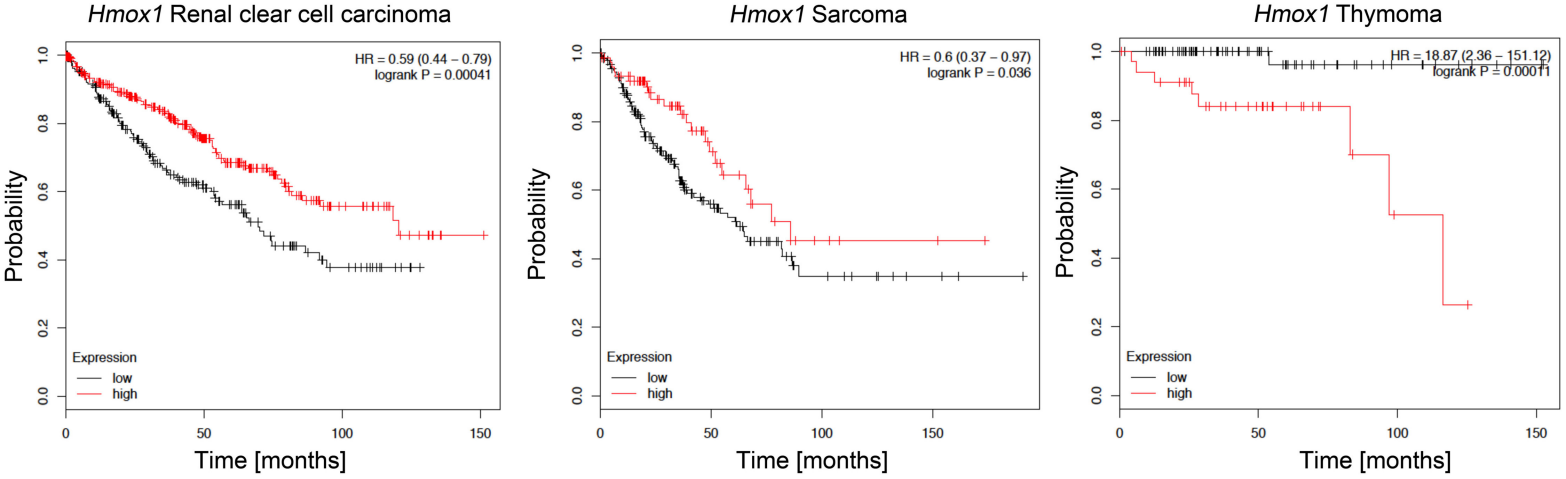
Kaplan-Meier survival plots for patients stratified by *HMOX1* expression. Kaplan-Meier plots were obtained by using the Kaplan-Meyer plotter online database tool (122). Patients were stratified for high and low expression of *HMOX1* in renal clear cell carcinoma, sarcoma, and thymoma patients.

HO-1 is normally expressed in the spleen and liver, but it can also be induced in many other organs/tissues by a variety of stimuli, including heme, ROS levels or hypoxia (118). In cancer, HO-1-overexpression has been reported in leukemia and in several solid tumors, including glioblastoma, melanoma, and hepatocellular carcinoma (119–121). Interestingly, both renal clear cell carcinoma and sarcoma patients with high *HMOX1* expression exhibited better survival rates than those with low *HMOX1* expression, while the opposite is true for thymoma patients, indicating the complexity of HO-1 biology in cancer [Figure 5, data based on kmplot.com; (122)] In many tumors HO-1 is suggested to act as a survival molecule, promoting cancer cell growth, metastasis, angiogenesis and resistance to chemotherapy (123, 124). However, HO-1 expression was found to be decreased in patients with early-stage non-small cell lung cancer (125) and its induction increased cell death and inhibited the migratory ability of hepatocellular carcinoma (HCC) cells (126, 127), suggesting tumor type specific effects. Specifically, HO-1 seems to hinder HCC progression via downregulation of miR-30d/miR-107 expression, a mechanism that also involves PI3K/AKT and MAPK/ERK pathways (128). HO-1 activity may help to mitigate DNA damage, gene mutation and carcinogenesis resulting from excessive ROS levels; we have previously showed that HO-1/CO facilitate DNA damage repair via ATM-γH2AX mechanism in normal cells (129). However, HO-1 was also shown to promote chemotherapy-induced cell death in cancer cells (130). There is also evidence that HO-1 activity can result in iron accumulation, which several epidemiological and experimental studies associate with increased cancer incidence and risk, tumor initiation, growth and metastasis (131, 132). Since iron is highly reactive and it continuously exchanges between its different oxidized forms, excess iron induces free radical formation, lipid peroxidation, DNA and protein damages, with important consequences in carcinogenesis (133). Emerging evidence has also revealed that HO-1-induced iron levels can impact ferroptosis, a form of oxidative cell death that plays a critical role in the pathogenesis of diseases involving iron overload, such as cancer (134). Recent data indicated a crucial role of tumor-associated macrophages (TAMs)-derived iron within the tumor microenvironment in disease progression, implying that HO-1 expressed in these cells plays profound roles in modulating tumor microenvironment and promoting metastasis (135). Of note, we showed that HO-1 in TAMs is critical for regulating epithelial-mesenchymal transition (EMT) and metastatic outgrowth in prostate cancer (136).

Although HO-1 is normally resident on the endoplasmic reticulum, it has been detected in the nuclei of prostate, lung, and oral cancer tumor cells, where it is correlated with tumor progression (137–139). However, the pathophysiological relevance of this finding and any specific mechanisms involved are not yet fully understood. It is well-known that HO-1 undergoes proteolytic cleavage with subsequent nuclear translocation under stress conditions *in vitro*, and this promotes tumor growth and invasion independently of its enzymatic activity (138, 140). Nevertheless, HO-1 lacks a DNA binding domain and whether it can interact with transcriptional control factors or chromatin proteins to impact the expression of specific genes related to cancer progression is still a matter of debate and deserves further investigation (141).

Importantly, HO-1 can also impact cancer progression by participating in both innate and adaptive immune responses in the tumor microenvironment. By suppressing the expression of proinflammatory cytokines (i.e., TNF-α) and promoting the expression of immunosuppressive cytokines (i.e., IL-10), HO-1 significantly modulates the immune regulatory functions of myeloid cells that control TME maintenance, for example by promoting inflammation-associated angiogenesis through up-regulation of VEGF expression in macrophages (141, 142). A more recent study showed that IL-6-driven expression of HO-1 in TAMs facilitated transendothelial migration and metastatic spread of breast cancer cells, suggesting that HO-1^+^ macrophages can significantly influence TAMs phenotype and cancer progression (143).

**Figure 6:**
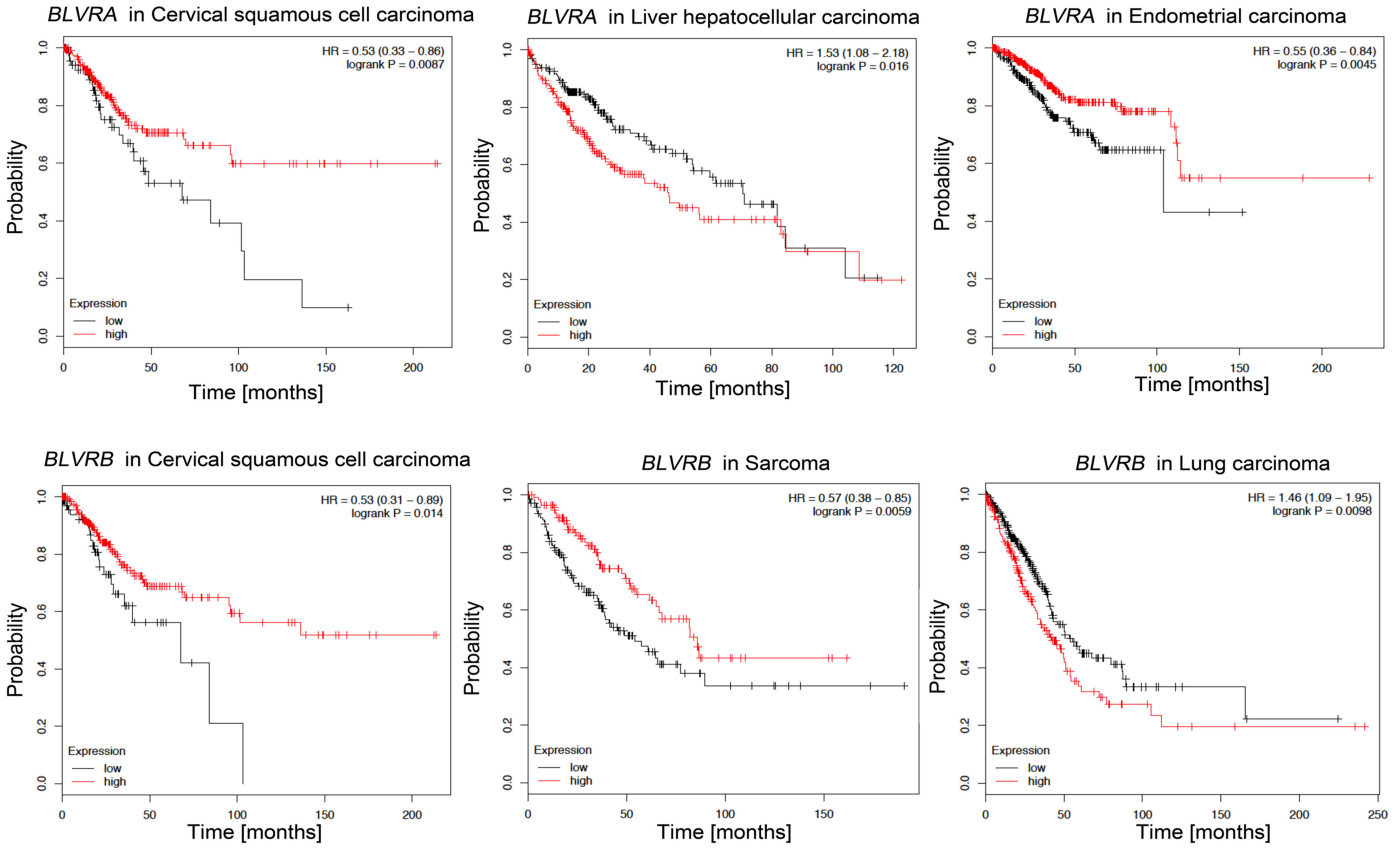
Kaplan-Meier survival plots for patients stratified by *BLVR-A* and *BLVR-B* expression. Kaplan-Meier plots were obtained by using the Kaplan-Meyer plotter online database tool (122). Patients were stratified for high and low expression of *BLVR-A* in cervical squamous cell carcinoma, endometrial carcinoma and liver hepatocellular carcinoma; and for high and low expression of *BLVR-B* in cervical squamous cell carcinoma, sarcoma, and lung adenocarcinoma.

Since various BLVR functions are related to signaling and gene expression that regulates cell growth, differentiation and survival, BLVR-A and BLVR-B may be directly involved in the development and progression of cancer (83). Supporting this, high levels of *BLVR-A* expression have been reported in malignancies including skin, breast, lung, and liver cancers (51, 144, 145), while *BLVR-B* is highly expressed in esophageal carcinoma, leukemia, and hepatocellular carcinoma (146–150). Importantly, *BLVR-B* was found to be most highly expressed at the tumor invasive margin in endometrial carcinoma, suggesting a role for this protein in cancer invasion (151). Indeed, BLVR-A has also been reported to affect the cell morphology and processes involved in EMT, a common characteristic of metastatic cancers (49, 51, 152). Additionally, a few studies have identified both BLVR-A and BLVR-B levels as potential biomarkers for the diagnosis, prognosis or treatment of prostate, pancreas, and vaginal carcinomas (153–155). Our analyses based on available gene expression data (kmplot.com) suggests that similar to HO-1 (above), there may be a role for BLVR in patient survival dependent on cancer type. High *BLVR-A* expression confers survival benefits in patients with cervical squamous cell carcinoma or endometrial carcinoma, while low expression levels are more beneficial for patients with liver hepatocellular carcinoma (Figure 6). High levels of BLVR-B expression in cervical squamous cell carcinoma and sarcoma patients is also correlated with better survival than those with lower *BLVR-B* expression. However, the opposite appears true for lung adenocarcinoma patients (Figure 6). Elevation of *BLVR-A* expression in tumor cells has been linked to cancer-associated hypoxia (83). Gibbs et al. showed that *BLVR-A* expression is driven by HIF1-α induction (156) and a similar mechanisms for regulation of BLVR-A levels in hypoxic conditions was seen in pulmonary arterial smooth muscle cell (PASMC) and human glioblastoma cells (157, 158). The role of BLVR in cancer is closely related to oxidative stress: the knockdown of *BLVR-A* resulted in a significant increase in intracellular ROS levels in glioblastoma cells and resulted in increased *HMOX-1* expression (50, 158, 159). In addition, nuclear BLVR-A acts as a transcription factor and binds directly to ARE/AP1 and ATF2/CRE DNA sequences or in complex with Erk1/2/Elk or Nrf2/ARE, affecting multiple signaling pathways involved in cancer progression (160). Finally, BLVR-A overexpression enhanced drug resistance and protection against chemotherapeutics (cisplatin and doxorubicin) (50, 158). Therefore, due to their regulatory effects on signaling and transcription, BLVR and its related bile pigments may impact both the development and progression of cancer.

While direct evidence for a role of BLVR in the TME has not been shown, there is indirect evidence for its influence on immunosenescence, polarization of macrophages and function of T cells in ways that are known to contribute to carcinogenesis. Several reports have demonstrated that elevated BR is toxic (161–163) and may damage erythrocytes and neurons and can induce cell death (164–167). BR can activate astrocytes and neurons to release soluble factors through MAPKs and NF-κB that ultimately reduce the production of tumor necrosis factor (TNF)-α, interleukin (IL)-1β, and IL-6 by microglia, which is important for the regulation of immunology within the central nervous system (168, 169). Furthermore, our recent data suggest that BV stimulates removal of cellular debris by macrophages and is required for the *HMOX1*-mediated protection against immunosenescence in myeloid cells (170). BR also has immuno-modulatory and anti-inflammatory properties through the induction of necrosis and apoptosis in mature immune cells (9, 171–173). In T helper type 17 (Th17) cells, BR exerts its immunomodulatory properties through AhR-dependent upregulation of CD39, with beneficial effects for patients with inflammatory bowel disease (IBD) (26). Moreover, mildly elevated BR levels modulate intracellular signaling pathways involved in immunosuppression and protect against diseases associated with increased oxidative stress (86, 160, 174–176). For example, exogenous BR supplementation suppressed DAMP release and altered cytokine profiles in a model of pancreatic islet transplantation, showing overall cytoprotective and antioxidant effects and suggesting that this method could be used to improve outcomes after allograft transplantation (8). In the same model, BR treatment of donors is known to lower the levels of iNOS and IL-1β and to increase islet quality, resulting in lower inflammatory response in the recipients, which also improves transplantation outcomes (177, 178). This suggests that targeting BR metabolism and/or BLVR enzymatic activity could have important consequences in the tissue microenvironment and could be a potential therapeutic approach for metabolic, cardiovascular, oncogenic and neurological disorders as well.

## Targeting Blvr In Cancer And Immune Diseases

Since BLVR plays a major role in cellular signaling and gene expression by promoting oxidative and immune homeostasis it is therefore considered a potential therapeutic target for a variety of diseases (66). Recent studies employed BLVR-based peptides to inhibit BLVR-A kinase activity or its interaction with growth-promoting kinases (83). Two human BLVR-A-derived peptides (FGFPAFSG and KKRILHCLGL) have been shown to inhibit the activity of Erk1/2 by blocking the formation of the complexes that include BLVR, MEK1 and Erk1/2 or BLVR, PKCδ and Erk1/2 (57, 58, 179, 180). Regulating the activity of BLVR and its ability to form such complexes may block the activation of Erk1/2 upstream, offering a novel approach to slow the growth of tumor cells with hyper-activated Erk1/2 or to treat any other inflammatory disease related to its activation (83, 181). Another BLVR-derived peptide (SFHFKSGSL) was described as a potent PKCδ inhibitor, with the ability to induce apoptosis by disrupting the cell membrane integrity (182). This further supports a potential therapeutic application of BLVR-based peptides in tumors showing excessive PKCδ activation, such as non-small cell lung cancer or breast cancer (58, 182–184). Two BLVR-derived peptides have been demonstrated to bind to the insulin receptor kinase (IRK) and alter its secondary structure, with important effects on glucose and insulin metabolism (185). Specifically, the KYCCSRK peptide was able to stimulate glucose uptake and to increase insulin function, while the KEDQYMKMTV peptide inhibited IRK activity and glucose uptake (185). BLVR is a substrate for IRK and it shares regulatory motifs and sequences with proteins that function in insulin and insulin-like growth factor-1 (IGF-1) pathways (48, 58, 82, 186). BLVR-A protein levels and activation are significantly reduced in peripheral blood mononuclear cells from obese patients and are associated with impaired insulin signaling, obesity, metabolic syndrome, NASH and visceral adipose tissue inflammation, indicating that BLVR-A modulation could be a therapeutic approach to obesity prevention and care (187). These peptides have been delivered successfully both *in vitro* and *in vivo*, supporting their potential as a novel therapeutic approach to control abnormal glucose metabolism and insulin resistance (185, 188–190). A major limitation of a peptide-based therapy is the short half-life of peptides in the circulation, which can result in a lower delivery compared to antibody-drug conjugates (191, 192). Hence, more effort needs to be made on improving the pharmacokinetics as well as the therapeutic efficacy of peptides or their derivatives *in vivo*. Alternative administration routes such as respiratory, local (intra-tumor injection) and site-specific delivery should also be considered (193). The use of enhancers or nano/micro particulate carriers also represents a promising approach for peptide-targeted delivery (194, 195). Interestingly, a recent report showed that bilirubin-based nanoparticles may represent a valid carrier option for many small peptides whose therapeutic efficacy is limited by their short circulation half-life (196) and this method could therefore be used for a more efficient delivery of BLVR-based peptides *in vivo*. Furthermore, BR-based nanoparticles have been tested in several diseases and conditions for their efficacy in tumor targeting and drug release (197–202). A second limitation of a peptide-based therapy is that BLVR kinase activities cannot be completely separated from its reductase activity and the use of BLVR-based peptides can affect both pathways simultaneously. Thus, the possible side effects that will likely compromise BLVR reductase activity in healthy cells need to be addressed. These effects might be more evident in macrophages or vascular endothelial cells, where BLVR has a significant reductase activity but also mediates homeostatic signaling regulating immune responses (47, 203). Such an issue highlights the importance of using peptide delivery systems capable of targeting specific cellular compartments to reduce the potential for systemic side effects.

In addition to peptides, BLVR-A modulation can also be achieved by using small molecules. In a recent study, several FDA-approved compounds were screened and tested for their effects on BLVR-A activity in the context of hyperbilirubinemia (204). After the initial characterization of safety profile and oral absorption, only two compounds were selected for further studies. However, one of them failed to reduce bilirubin levels, while the other proved to be hepatotoxic in rats (204). More BLVR-A inhibitors could be evaluated for their structure-activity relation to produce even more potent inhibitors. Moreover, the availability of the *BLVR-A* conditional and total knockout mice will further help dissect the role of this pathway in various diseases. The manipulation of BLVR can also be an attractive strategy to overcome treatment-related multidrug resistance, a serious concern in patients treated with chemotherapy, in whom the tumor cells develop resistance to multiple classes of chemotherapeutic agents (205). A few studies showed that overexpression of *BLVR-A* can enhance multidrug resistance, while its inhibition can overcome multidrug resistance and re-establish drug sensitivity (50, 158, 206).

## Concluding Remarks

In conclusion, heme catabolism and its resulting products exert significant control of both homeostatic and pathophysiologic processes. BLVR-A and BLVR-B are multifunctional proteins with activities ranging from enzymatic activity to signaling kinases and regulators of transcription. In conjunction with HO-1 and heme metabolites, BLVR provides a cytoprotective and immunomodulatory mechanism for the cell and the host. Therefore, it is pivotal to understand the molecular mechanisms regulating heme metabolism for the maintenance of homeostasis and the impact of these proteins as drivers in various immune pathologies. Similarly, increased awareness of the role heme metabolites play and their modes of regulation in response to various stressors will likely prove to be extremely useful in the treatment of several pathologies.

## Author Contributions

GC and SH wrote a first draft. KS and BW edited the text. GC and BW prepared the figures.

### Conflict of Interest

The authors declare that the research was conducted in the absence of any commercial or financial relationships that could be construed as a potential conflict of interest.
